# Risk messaging style and its effect on public preparedness for earthquakes: longitudinal intervention-based study

**DOI:** 10.1057/s41271-024-00534-w

**Published:** 2024-12-02

**Authors:** Liel Levy, Moran Bodas

**Affiliations:** Department of Emergency & Disaster Management, School of Public Health, Faculty of Medical and Health Sciences, Tel-Aviv University, Tel-Aviv-Yafo, Israel

**Keywords:** Earthquake preparedness, Risk communication, Fear tactics, Empowerment

## Abstract

This study examines the effect of risk communication styles (fear-based versus empowerment-based) on households’ earthquake preparedness. An online longitudinal study with intervention and control groups was conducted using a representative sample of the adult population in Israel. The change in the reported level of preparedness was assessed through Repeated Measures ANOVA with interaction effects for both the risk communication style and gender. The Analysis revealed a significant difference in reported levels of earthquake preparedness over time (F(1.697,303.70) = 102.58, *p* < 0.001; partial *η*2 = 0.36). However, no statistically significant interaction was found with the risk communication style (*p* = 0.55). Borderline significance (*p* = 0.04) was observed in the three-way interaction (time-intervention-gender). Gender (*β* = 0.19), age (*β* = 0.21), perceived earthquake likelihood (*β* = 0.14), and sense of preparedness (*β* = 0.28) were significant predictors in multivariate regression analysis. While consistently showing that participants exposed to empowering information reported higher earthquake preparedness, the research hypothesis was not substantiated. Recommendations for public health policy are discussed.

## Key message

- Inadequate levels of household emergency preparedness may be due to ineffective risk communication, which fails to motivate actual preparedness.
- This study examined the promotion of emergency preparedness behavior through innovative, empowerment-based risk communication compared with fear-based messaging.
- Participants exposed to empowering information reported higher earthquake preparedness at all measurement points, although not statistically significant from the fear-based messaging group. This result might be due to the participants in the fear condition also perceiving that condition as empowering.

## Introduction

Emergencies and disasters take a heavy toll globally each year. Research analyzing trends in the occurrence of emergencies and disasters indicates a continuous increase in the number of reports related to natural disasters (such as earthquakes, floods, and pandemics) and human-made disasters (such as war and terrorism) over the years [1, 2].

According to the literature, populations that are better prepared for emergencies and disasters cope more effectively, experience fewer casualties, and recover at a faster rate [3–6]. Strengthening public preparedness is considered a crucial component for an efficient response from both the public and the government in emergencies, as well as for recovery and reconstruction thereafter [7, 8]. Adequate emergency preparedness can significantly mitigate the negative impacts of disasters on countries, ensuring that citizens can take care of themselves and their families at least during the initial 72 h post-disaster, before external assistance arrives [5, 9].

Even though the magnitude and frequency of these events are increasing, a sufficient level of preparedness among populations for emergencies has not been achieved [10–13]. Promoting public preparedness for emergencies poses a challenge for many countries [14–16]. In Israel, it has been found that the level of preparedness for various types of disasters is disasters is similarly suboptimal—approximately 50% in a war scenario [17] and approximately 35% in an earthquake scenario [8].

Emergency preparedness at the household level entails several elements. These include having provisions or stockpiles of essential goods, such as canned or wrapped food items, bottled water, first aid kit, flashlight. [7, 18, 19]. In addition, family members ought to be aware, informed, and educated about the threats relevant to their area [20, 21]. For earthquakes, in particular, household adjustments are needed to secure heavy furniture to walls, remove objects from shelves hanging over beds or sofas, and identify safe routes to exit buildings in the shortest possible time [5, 6, 22]. Prior research demonstrated several correlates of household adjustment behavior for earthquakes including prior experience [23, 24], seeking relevant protection information [5, 7, 22, 25], social trust in authorities [26, 27], threat perception [28], perception of responsibility and self-efficacy to perform suggested actions [28–30], and different socio-demographic variables, including gender and age [5, 28]. The literature also accounts for the governing effect of fear and appraisal of control over earthquake preparedness behavior [31]. In fact, the scientific literature suggests that anxiety, in particular trait anxiety, is negatively associated with disaster preparedness behavior in general [32–35].

Arguably, one of the reasons for observing low or inadequate levels of household emergency preparedness may be ineffective risk communication, which fails to motivate actual preparedness [36]. Risk communication about health-promoting behaviors, including emergency preparedness, primarily relies on the assumption that the public suffers from an information deficit [37]. It implies that exposure to additional information related to risks will increase threat perception and encourage preparedness [38–40]. However, this assertion is challenged by the reality that there is an abundance of campaigns promoting emergency preparedness, yet the actual preparedness remains low [40].

Moreover, research suggest another possible explanation for the short-comings of disaster risk communication campaigns—Fear appeal. Many risk communication campaigns rely on the assumption that by vividly demonstrating the negative consequences of undesirable behavior, individuals will reduce their risky behavior and adopt alternative, safer behaviors [38]. While fear-inducing content may contribute to short-term engagement in preparedness for hazards, this influence is usually short-lived [41, 42].

In light of the above, the importance of finding innovative approaches to risk communication that do not rely on fear tactics is evident. Preliminary evidence for such innovative approaches was observed during the global response to the COVID-19 pandemic, when a need to transition from risk and self-protection messages to messages based on altruism and social solidarity was reported [43–47]. Furthermore, recent research published in the scientific literature suggests that messages of empowerment and optimism have some ability to promote sustainable behavioral change, especially concerning emergency preparedness [48–50].

This study aims to examine the possibility of promoting emergency preparedness behavior by risk communication using an innovative approach based on empowering and optimistic messages. The primary research hypothesis is that such an approach to risk communication will enable those exposed to it to achieve the desired behavioral change, compared to risk communication based on fear-appeal.

## Data and methods

### Study design and procedure

The study was conducted as a prospective longitudinal study with intervention and control groups using questionnaires which took place during the months of August–October 2023. The first data collection point was during 3–10 August 2023. The second data collection point was two weeks later between August 28 and September 5, 2023. The third data collection point coincided with the first week following the October 7th attacks in Israel and took place between October 14 and 22, 2023.

Participants were recruited for the study from an online panel called SekerNet (sekernet.co.il). The company follows the European Society for Opinion and Market Research (ESOMAR) guidelines to uphold research standards. Participants were prompted to enter links to fill in the questionnaire at the same time. Data collection followed pre-determined quotas for gender, age, and geographical distribution. Participants were randomly allocated to the study groups: (1) 100 participants in the control filled out baseline questionnaires measuring, received an earthquake information leaflet by the Israeli Civil Defense, and did not got through any further intervention. (2) 350 participants in the fear-appeal group filled out the baseline questionnaire and got the leaflet, but also watched a preparedness video produced by the Israeli Civil Defense on earthquake preparedness, which was laced with fear tactics. The video starts with images of shaking buildings and destruction, a black and red color scheme, dramatic music, and messages about the earthquake risk in Israel. It describes past deadly earthquakes and indicates that the next one is not a question of if, but when. Later, the video transitions into a more relaxed audio-visual package and provides a detailed description of all the recommended actions to be taken by a household in preparation for a major earthquake. (3) 350 Participants in the empowerment group followed the same approach, but instead watched a video developed ad hoc for this study featuring a female, mother protagonist transitioning from a hopeless parent during a light earthquake to a goal-oriented mother determined to prepare for the next earthquake. Through an upbeat, inspirational audio-visual package, the video illustrates the recommended actions to be taken by a household in preparation for a major earthquake. The video shows the mother’s journey in passing forward the information to others, indicating the fact that most survivors following a major earthquake are extricated by laypersons, therefore everyone can take part in saving lives.

### Study population and sample

The study population is the adult population (aged 18 and over) in Israel, in a representative sample according to quotas of gender, age, geographical distribution, and education. The population included participants from different seismic risk areas according to the Inter-Ministerial Steering Committee for Earthquake Preparedness. Inclusion criteria: Adult (aged 18 and over), Hebrew speaker. Exclusion criteria: Minor (under 18 years of age), non-Hebrew speaker.

Minimum sample size calculation was based on data retrieved from a study conducted by Tanes et al. (2011) [51], which was the closest study in design to the current study. The authors reported an average difference of 0.84 points between the intervention group, which used earthquake preparedness computer games, and the control group. According to the OpenEpi calculator [52], in order to achieve this average difference in the current study with a confidence level of 95% and a power of 80%, 52 participants were required. However, to ensure a diverse and representative sample and to cope with the expected dropout of participants between the different stages of the study, a total sample of 800 participants was sampled.

### Variables and tools

The main independent variables is the type of risk communication (none, fear-appeal, and empowerment-based). The main dependent variable in this study is population preparedness for an earthquake, which is measured by the Preparedness Index (PI) [17]. This index describes the number of actions that respondents report having taken to prepare their home for the event of an earthquake, out of a list of 12 recommended actions for adapting households to earthquakes. Respondents were asked to indicate whether they had performed the various actions during the past five years using a binary response (yes/no). The PI score is calculated as the sum of the number of actions for which the respondent answered ‘yes,’ with each item having equal weight in the calculation of the final score, as is customary in research in this field [17, 28]. The PI is a continuous variable with values from 0 (no preparedness at all) to 12 (full preparedness). In the current study, the internal consistency (Cronbach’s alpha) of this scale was.858. This scale was assessed in all three data collection points.

Additional independent and confounding variables were assessed, including:

*Risk Perception*: Measures of perceived likelihood (2 items—one for the next year and another for the next 5 years), severity (5 items—assessing severity to the society, community, participant’s routine, participant’s belongings, and participant themselves), and intrusiveness of the threat (1 item assessing risk personalization—see Mileti & Sorensen, 1990 [53], for a historical review and Fischer et al. 2023 [54], for a more recent discussion of this variable). Adapted from Bodas et al. (2015) [17] with a Cronbach’s alpha values of 0.854. It is measured using a five-point Likert scale questionnaire (from 1 "not at all" to 5 "very much"). Example item: "What do you think is the likelihood of a strong earthquake next year?" This scale was assessed once before the intervention.

*Trait Anxiety*: Developed to measure an individual’s tendency to experience anxiety [55]. Adapted and validated in Hebrew by Popper et al. (2014) [56], comprising 20 items on a 4-point Likert scale (1- not at all, 2- slightly, 3- moderately, 4- very much). Higher scores indicate a higher level of trait anxiety. In the current study, the internal consistency (Cronbach’s alpha) of this scale was 0.928. This scale was assessed once before the intervention.

*Sense of Preparedness*: this measure was calculated as the mean response score to four items on a five-point Likert scale and adapted from Bodas et al. (2015) [17]. The items included in the questionnaire are: (a) "To what extent are you familiar with the Home Front Command’s recommendations for earthquake preparedness?", (b) "To what extent are you emotionally/mentally prepared for an earthquake?", (c) "To what extent do you know what to do in the event of a major earthquake?", and (d) "To what extent do you feel physically prepared for an earthquake?". Participants were asked to give an assessment of these questions before reporting on their actual actions to adapt their household to an earthquake. In the current study, the internal consistency (Cronbach’s alpha) of this scale was 0.801. This scale was assessed in all three data collection points.

*Trust in Authorities*: Perception of trust ("rank the extent of trust you have in the following:") was measured using a set of eight-item questionnaires on a 5-point Likert scale (1- not at all, 5- very much) Trust was assessed for the government, the civil defense authorities, local authorities, first responders, media, politicians, local aid organizations, and the health system. This questionnaire was translated into Hebrew and validated in the study by Bodas et al. (2022) [57]. In the current study, the internal consistency (Cronbach’s alpha) of this scale was 0.756. This scale was assessed once, before the intervention.

*Perception of Responsibility*: Perception of preparedness responsibility ("to what extent do you think it is the responsibility of each of the following to prepare for a major earthquake?") was assessed using six-item questionnaire on a 5-point Likert scale (1- not at all, 5- very much) to explore assigned responsibility for earthquake preparedness on the government, the civil defense authorities, the local authority, the health system, the community, and the participant and their family members. This questionnaire was translated into Hebrew and validated in the study by Bodas et al. (2022) [57]. In the current study, the internal consistency (Cronbach’s alpha) of this scale was 0.836. This scale was assessed once, before the intervention.

*Prior Exposure to Earthquake*: Participants were asked to indicate whether they had experienced a major earthquake in the past on a dichotomous scale (yes/no). A definition of what constitutes a strong earthquake was given to increase uniformity in the participants’ responses. This scale was assessed once, before the intervention.

*Seismic Threat Level*: The seismic risk area (level) was defined according to the Inter-Ministerial Steering Committee for Earthquake Preparedness and was a continuous variable on a scale of 0 to 10, with 0 indicating a low seismic intensity coefficient and 10 indicating the highest, according to the national standard map. For each participant, a seismic risk index was assigned according to the settlement of residence, which they indicated in the questionnaires. The higher this index, the more the area of residence is defined as an area with increased seismic activity and therefore a higher seismic risk. This scale was assessed once, before the intervention.

*Socio-Demographic Variables*: Gender (2 categories), age (continuous), education (ordinal, 5 categories), income (ordinal, 5 categories), and number of children under 18 (continuous). These variables were assessed once before the intervention.

In addition, the questionnaire included manipulation check items measured using a five-point Likert scale to assess participants’ perception of the intervention as either scary, optimistic, and empowering. Lastly, the questionnaire also assessed the motivation to engage in earthquake preparedness behavior on a five-point Likert scale.

### Tool validation

Although most of the research instruments used in this study were validated in previous studies, a preliminary pilot study was conducted with 30 participants from the target population to examine the internal consistency of the research instruments. In addition, to examine the characterization of the intervention videos used in this study, a two-round expert panel was conducted. The panel was conducted using the electronic Delphi method [58]. Experts were asked to review characterizations of fear-appeal and empowerment risk messaging, as well as to review the videos selected for the study, and express their opinions on their appropriateness for the study.

### Statistical analysis

Statistical analysis of the results was performed using SPSS version 28. First, the relevant measures of central tendency and dispersion were presented for all variables at the first point of the study. Before performing the statistical analysis, the normality of the distribution of the variables was tested using the Kolmogorov–Smirnov test. The reliability of the measures was tested using Cronbach’s Alpha index. Correlations between continuous variables were computed using Spearman’s rank order correlations. The relationship between categorical variables was tested using a Chi-Square. Differences in means between categories were tested using an independent samples T-test or ANOVA. Repeated Measures ANOVA was used to estimate the effect of the intervention on the PI, controlling for gender. A multivariate linear regression was performed to predict the preparedness index. The analysis was performed using the forward selection procedure for stepwise regression with predictor variables that were found to be related to the dependent variable in the univariate analysis. The analysis was performed after verifying the assumptions of the model, including the absence of multicollinearity, and testing for homoscedasticity**.** In all statistical analyses, a *p*-value of 0.05 was the threshold for statistical significance, except in cases where a correction was made for multiple comparisons.

### Data quality assurance

Prior to data analysis, a cleaning process was performed to ensure that the file included quality responses that met the pre-defined research criteria. Two criteria for data inclusion were applied. The first was response time, i.e., excluding participants who took too long or too little time to respond with a tolerance of one standard deviation from the mean time. The second was reported readiness (the Preparedness Index). Since a decrease in readiness is less likely and may indicate bias or reporting bias, a criterion was established stating that a decrease of up to one action from round to round would be tolerated. The final sample included 401 respondents (about a half of the original dataset). Of the 404 excluded participants, 107 (~ 26%) were removed based on the reported readiness criterion, and the remainder based on time spent on filling out the questionnaire. To ensure that participant exclusion did not affect the socio-demographic distribution of the sample and that final sample was representative of the total sample, differences between the final sample and the total sample received from the survey company were examined (see Table 1). In addition, analysis was conducted to ensure that dropouts were not statistically different in reported Preparedness Index compared with the final sample (data not shown).

**Table 1:** Distribution (*N*, %) of socio-demographic variables of the different samples

Variable	Final sample (*N* = 401)	Sample at *t* + 14 days (*N* = 656)	Sample at *t* + 60 days (*N* = 533)	Total sample (*N* = 805)
Gender				
Female	204 (50.9%)	312 (47.6%)	247 (46.3%)	395 (49.0%)
Male	197 (49.1%)	344 (52.4%)	286 (53.7%)	411 (51.0%)
Age	Mean 41.43, SD 16.39			
18–35	173 (43.2%)	236 (36.0%)	173 (32.5%)	319 (39.6%)
36–50	112 (27.9%)	186 (28.4%)	151 (28.3%)	230 (28.5%)
51–69	93 (23.2%)	190 (29.0%)	172 (32.3%)	212 (26.3%)
70 +	23 (5.7%)	44 (6.7%)	37 (6.9%)	45 (5.6%)
Level of religiosity				
Secular	183 (45.6%)	318 (48.5%)	252 (47.3%)	380 (47.1%)
Traditional	117 (29.2%)	203 (30.9%)	168 (31.5%)	248 (30.8%)
Religious	58 (14.5%)	75 (11.4%)	62 (11.6%)	100 (12.4%)
Orthodox	43 (10.7%)	60 (9.1%)	51 (9.6%)	78 (9.7%)
Number of children	Mean 1.8, SD 1.89			
0	144 (35.9%)	224 (34.1%)	174 (32.6%)	287 (35.7%)
1	48 (12.0%)	63 (9.6%)	56 (10.5%)	81 (10.0%)
2–3	148 (36.9%)	266 (40.5%)	218 (40.9%)	317 (39.3%)
4+	59 (14.7%)	103 (15.7%)	85 (15.9%)	119 (14.8%)
Missing	2 (0.5%)	0 (0.0%)	0 (0.0%)	2 (0.2%)
Education				
Elementary	12 (3.0%)	12 (1.8%)	9 (1.7%)	16 (2.0%)
High school diploma	117 (29.2%)	158 (24.1%)	125 (23.5%)	207 (25.7%)
Vocational	99 (24.7%)	174 (26.5%)	147 (27.6%)	213 (26.4%)
Academic degree	173 (43.1%)	312 (47.6%)	252 (47.3%)	370 (45.9%)
Income				
Much below average	89 (22.2%)	135 (20.6%)	107 (20.1%)	173 (21.5%)
A little below average	47 (11.7%)	91 (13.9%)	77 (14.4%)	113 (14.0%)
Same as average	113 (28.2%)	182 (27.7%)	143 (26.8%)	224 (27.8%)
A little above average	100 (24.9%)	163 (24.8%)	133 (25.0%)	195 (24.2%)
Much above average	30 (7.5%)	58 (8.8%)	49 (9.2%)	64 (7.9%)
Refuses to answer	22 (5.5%)	27 (4.1%)	24 (4.5%)	37 (4.6%)
Place of residence (dialing zones)				
Greater Jerusalem (02)	47 (11.7%)	80 (12.2%)	63 (11.8%)	102 (12.7%)
Tel Aviv and Center (03)	155 (38.7%)	243 (37.0%)	202 (37.9%)	302 (37.5%)
Haifa and North (04)	86 (21.4%)	142 (21.6%)	115 (21.6%)	172 (21.3%)
South and Coastal Plains (08)	70 (17.5%)	115 (17.5%)	92 (17.3%)	139 (17.2%)
Sharon region (09)	43 (10.7%)	76 (11.6%)	61 (11.4%)	91 (11.3%)
Seismic risk rating by residential area				
0.075–0.1	246 (61.3%)	398 (60.7%)	324 (60.8%)	487 (60.5%)
0.125–0.15	74 (18.4%)	131 (19.9%)	108 (20.3%)	165 (20.4%)
0.175–0.2	70 (17.5%)	108 (16.5%)	83 (15.5%)	130 (16.2%)
0.225 or above	11 (2.8%)	19 (2.9%)	18 (3.5%)	24 (2.9%)
Study group				
Control group	35 (8.8%)	80 (12.2%)	59 (11.1%)	100 (12.4%)
Fear-appeal group	195 (48.6%)	295 (45.0%)	242 (45.4%)	353 (43.8%)
Empowerment group	171 (42.6%)	281 (42.8%)	232 (43.5%)	353 (43.8%)

There are no statistically significant differences in any of the variables between the sample groups (*p* > 0.05 in all analyses), except for age (*p* = 0.04)

### Ethical considerations

The research was approved by the Ethics Committee of Tel-Aviv University before the commencement of the study (Approval Number: 0005479-1). Informed consent was obtained from all participants before any psychological intervention was conducted. Participants’ anonymity was always ensured, as the data was collected through a certified third party (survey service).

## Results

### Sample description

In total, the final sample comprised 401 Jewish participants. Table 1 illustrates the distribution of socio-demographic variables in the final sample compared to the raw data obtained from the survey company. Transitioning from the first measurement point to the second, the dropout percentage of respondents was 32.9% (*N* = 269). This percentage increased to 50.6% (*N* = 198) at the third measurement point.

### Descriptive findings

Close to 40% of respondents believed the probability of a strong earthquake in the next year was moderate. A similar percentage (36.6%) believed the probability was low or very low, while the remaining 23.5% believed it was high or very high. When asked about the probability of a strong earthquake in the next 5 years, almost 46% of respondents indicated a high or very high probability.

**Fig. 1:**
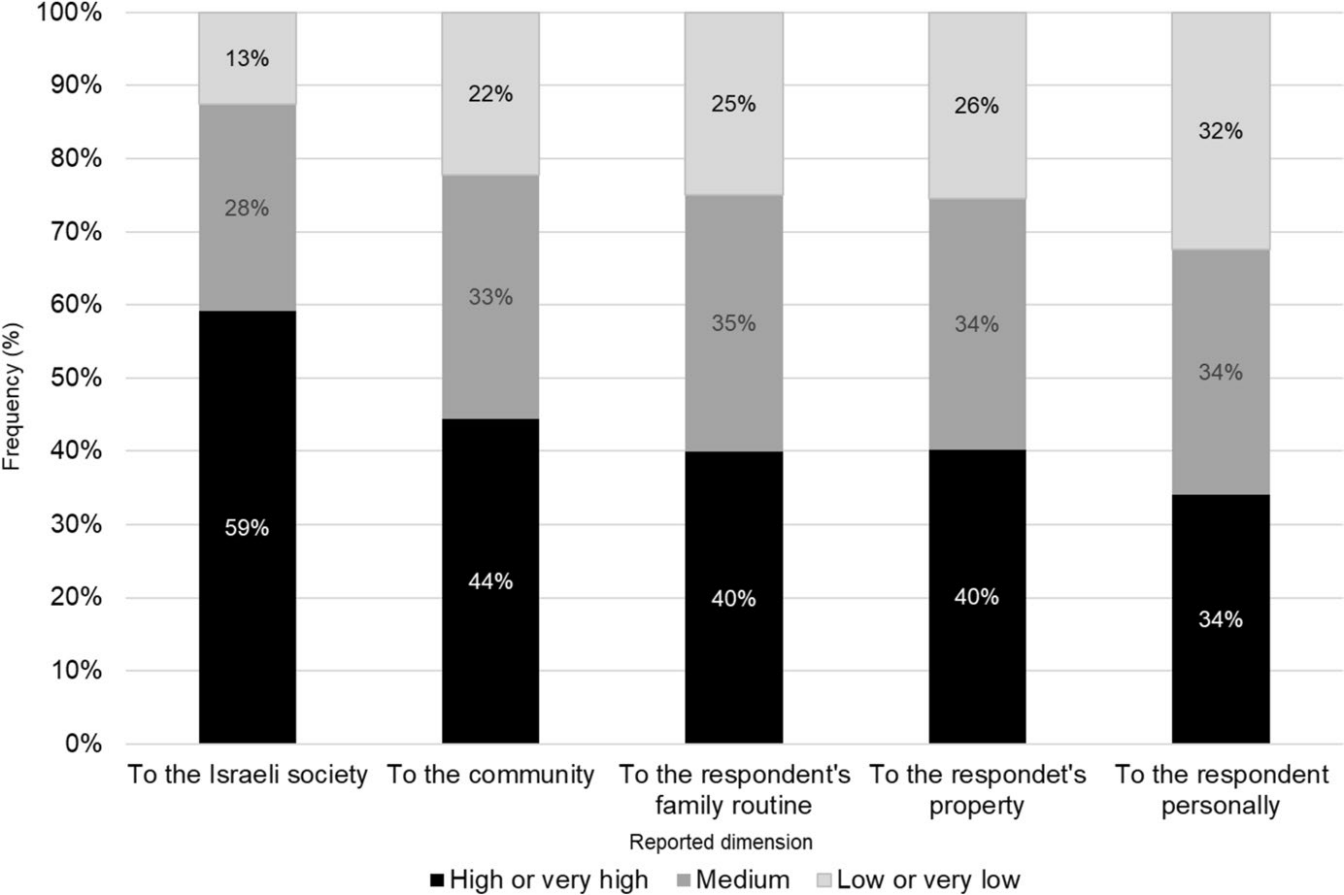
Distribution (%) of perception of earthquake severity according to different dimensions (*N* = 401)

As for the perceived intrusiveness of the threat, 46.1% of respondents indicated a moderate probability that they or their family would be affected by a strong earthquake, 42.7% indicated a low or very low probability, and 11.2% indicated a high or very high probability. In terms of perceived severity of damage, respondents tended to estimate the damage as lower the closer the dimension was to them personally (see Fig. 1).

In the final sample (*N* = 401), only 30 respondents (7.5%) reported having personally experienced a strong earthquake. 352 respondents (87.8%) answered no, and the rest (4.7%) answered don’t know/remember.

The respondents’ sense of preparedness changed over time. At the first time point, the mean perceived preparedness index was 3.05 ± 0.79SD, which was not statistically different than the measurement at the second time point (3.13 ± 0.78SD), according to paired samples t-test (*t* = 1.41, df = 268, *p* = 0.159). Similarly, between the second time point and at the third time point (3.22 ± 0.81SD) the increase was not statistically significant (*t* = 0.83, df = 197, *p* = 0.410). Nevertheless, the change between the first and third time point was marginally statistically significant (*t* = 2.06, df = 197, *p* = 0.041). Respondents were asked to indicate their level of trust in various authorities. Emergency and rescue organizations (71%) and the Home Front Command (58%) received the highest level of trust, followed by Community aid organizations (48%), the healthcare system (33%), the local authority (28%), the government (14%), the media (8%), and lastly the politicians (3.5%). In addition, respondents were asked to indicate who they believe is responsible for preparing for a strong earthquake. The results of this analysis demonstrate that the respondents believe that the Home Front Command bears the greatest responsibility for earthquake preparation (83.5% answered "to a great extent" or "very much"), followed by the local authority (69.4%), the government (60.9%), the health system (57.3%), the respondent or his/her family (46.9%), and finally the respondent’s community (33.9%).

Lastly, participants were asked to report whether the intervention video they watched was perceived by them as optimistic, empowering, and scary. Similar proportions of participants in the intervention groups perceived the video as "much" or "very much" empowering (59% in the empowerment video group and 53% in the fear-appeal group; *χ*^2^ = 2.80, df = 4, *p* = 0.592), In contrast, 45% of participants in the fear-appeal group perceived it as "much" or "very much" scary compared with only 8.1% in the empowerment group (*χ*^2^ = 98.75, df = 4, *p* < 0.001). Similarly, while only 18% of participants in the fear-appeal group perceived the video as "much" or "very much" optimistic, close to 55% of participants in the empowerment group thought it was optimistic (*χ*^2^ = 63.72, df = 4, *p* < 0.001). As far as motivation goes, similar proportions of respondents reported feeling "much" or "very much" motivated to engage in preparedness behavior after watching the videos (54% in the empowerment group and 60% in the fear-appeal group; *χ*^2^ = 5.43, df = 4, *p* = 0.246).

### Main outcome – earthquake preparedness index

**Fig. 2:**
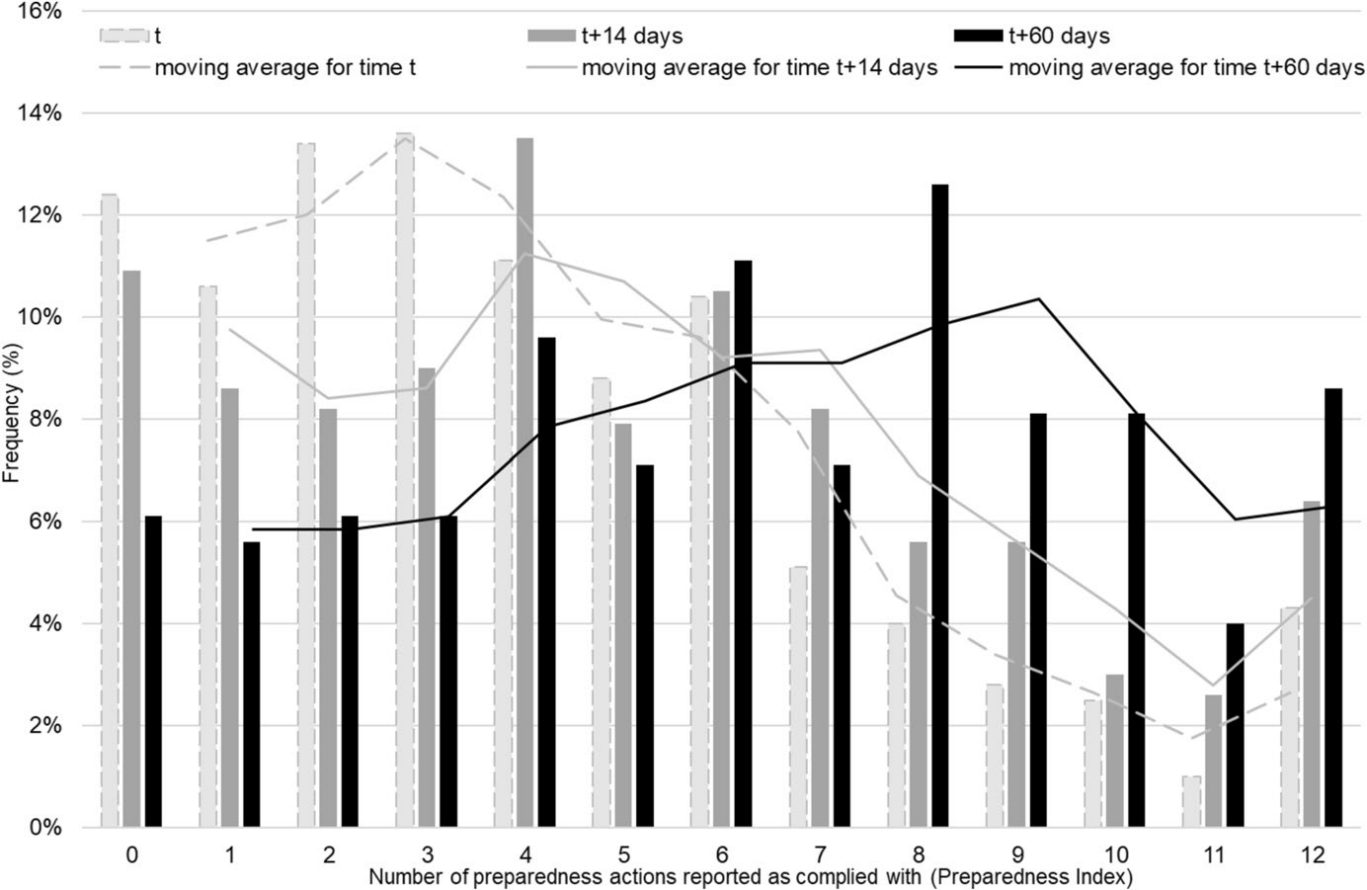
Distribution (%) of the preparedness index over time

The main dependent variable in the study is the Preparedness Index (PI). Table 2 details the distribution measures of this index over time. Table 3 provides the breakdown of the PI for each individual recommendation. Figure 2 provides the histograms of the preparedness index at the three measurement time points.

**Table 2:** Distribution of the earthquakes preparedness index over time

Measurement point	*N*	Mean	SD	Median	Inter-Quartile Range
First measurement point (*t*)	401	4.10	3.17	3.5	2.0–6.0
Second measurement point (*t* + 14 days)	269	4.97	3.46	4.0	2.0–7.0
Third measurement point (*t* + 60 days)	198	6.28	3.50	6.0	4.0–9.0

**Table 3:** Frequency (%) of compliance with household earthquake preparedness actions recommended by the civil defense authorities over time

Earthquake preparedness actions	1st time point (*t*) (*N* = 401) (%)	2nd time point (*t* + 14 days) (*N* = 269) (%)	3rd time point (*t* + 60 days) (*N* = 198) (%)
Know where and how to turn off water taps, gas, and main electricity switch	75.6	77.5	84.6
Place heavy items in low places, for example, large pot	57.5	65.6	71.4
Determined safe places in the house, where you can take shelter during an earthquake	47.7	60.5	67.5
Equipped with a flashlight and suitable batteries	40.1	47.9	66.8
Have acquired dry food and water in sufficient quantity for prolonged durations (several days)	23.5	29.2	57.4
Equipped with a first aid kit and medicines	30.2	39.4	52.3
Removed and put away anything that hangs over beds and sofas on which people sit or lie down	36.5	40.9	51.9
Hang mirrors and pictures with suitable hooks (as opposed to using only a nail)	28.5	39.8	48.1
Tightened loose fixtures and fixed tall furniture to the wall, such as a cupboard or a tall chest of drawers	29.0	36.9	46.2
Prepared a family emergency plan and made sure that all family members know it	18.8	30.4	39.5
Equipped with a battery-operated radio	21.6	23.1	38.9
In the kitchen, secured the closing of the doors of storage units, for example a cupboard where there are cups and plates are, to prevent them from falling out during a shake	22.4	29.0	32.6

To assess the change in reported earthquake preparedness level over time between the different research groups, a Repeated Measures ANOVA was conducted on the preparedness measure across three measurement points, cross-referenced with the research group (empowerment vs. fear), and controlled for gender. The test results indicate that it meets the assumption of equality of coefficients between the groups (Box’s *M* = 26.19, *F* = 1.41, *p* = 0.115). In contrast, Mauchly’s test for sphericity yielded a significant result (*χ*^2^ = 35.06, df = 2, *p* < 0.001). Therefore, the results were interpreted using the Greenhouse–Geisser degrees of freedom correction.

**Fig. 3:**
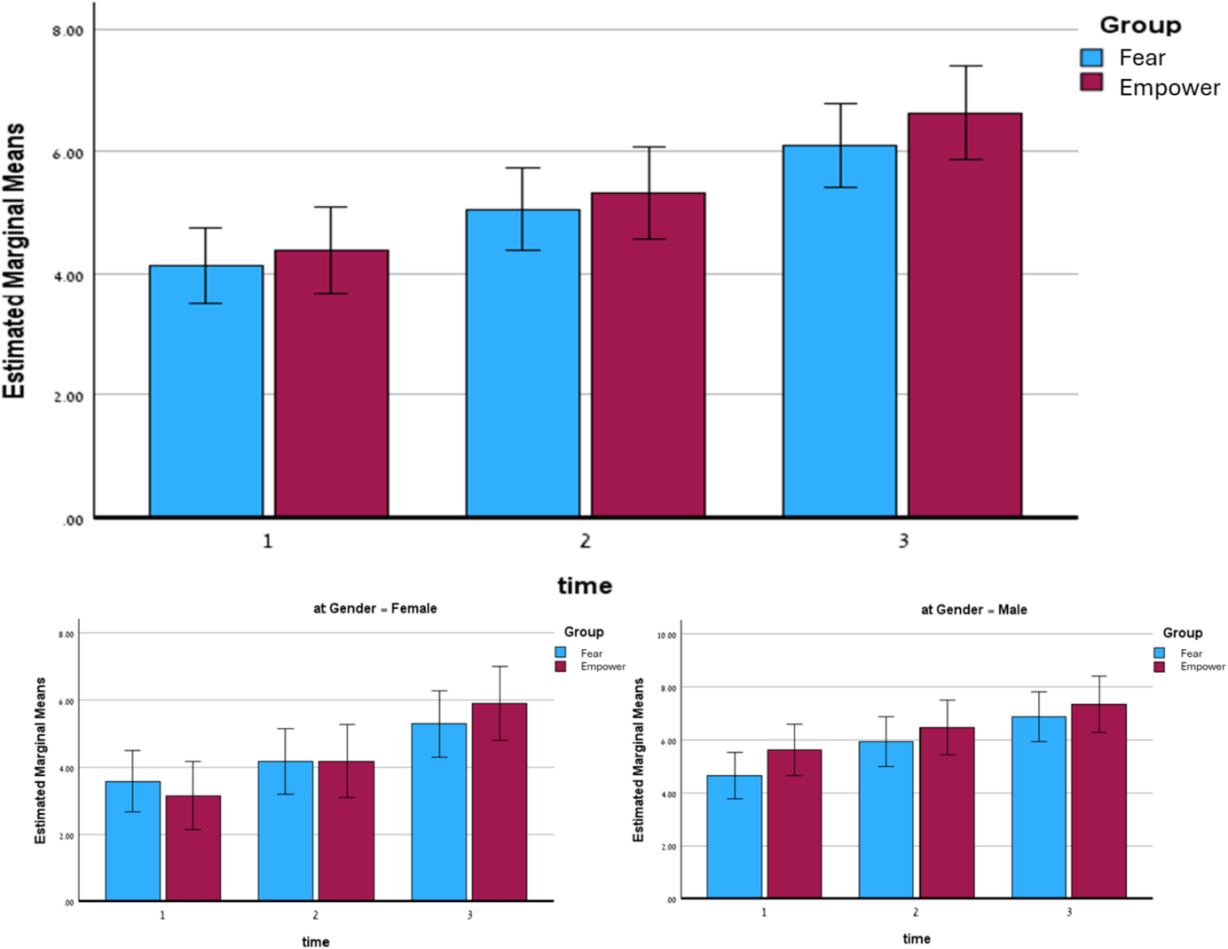
Results of repeated measurements ANOVA for assessing reported earthquake preparedness averages over time, grouped by research category: general sample (top image), among women only (bottom left image), and among men only (bottom right image). The margin of error (two standard errors) is depicted using bars

The findings of the Repeated Measures ANOVA analysis show a significant difference in reported earthquake preparedness level over time (F(1.697,303.70) = 102.58, *p* < 0.001; partial *η*^2^ = 0.36). No statistical significance was found in the interaction of time with research group (F(1.697) = 0.55, *p* = 0.551; partial *η*^2^ < 0.01), nor in the interaction of time and gender (F(1.697) = 1.49, *p* = 0.228; partial *η*^2^ < 0.01). However, the interaction of time with research group and gender was statistically significant (F(1.697) = 3.46, *p* = 0.040; partial *η*^2^ = 0.02), although it should be noted that this level of significance is not high enough for interactions of this type. Figure 3 summarizes the main findings of this section.

### Multivariate analysis to predict earthquake preparedness

Prior to performing the multivariate analysis, the correlates of the Preparedness Index were assessed. Table 4 provides the correlates of the PI at time *t*. Females reported lower preparedness (3.67 ± 2.99SD) than males (4.54 ± 3.30SD), according to independent samples t-test (*t* = −2.73, df = 394, *p* = 0.007). Participants who report to have experienced a major earthquake report higher preparedness (6.34 ± 3.87SD) than those without experience (3.89 ± 3.03SD) (*t* = 3.33, df = 30.93, *p* = 0.002). No difference in preparedness level (PI) was found according to religiosity (*p* = 0.988), education (*p* = 0.287), income (*p* = 0.591), and geographic location (*p* = 0.482), according to Spearman correlation. Similar results were obtained for the Preparedness Index at time *t* + 14 days and *t* + 60 days. The main difference at the second time point is the lack of a significant relationship between reported preparedness and the perception of the video as empowering (*p* = 0.066). The main difference at the third time point is the lack of a significant relationship between reported preparedness and the perception of the video as empowering, trait anxiety, or trust in authorities (*p* > 0.05) in both.

**Table 4:** Correlation analysis for the Preparedness Index at time "*t*"

Correlate	Spearman’s r
Age	0.22^***^
Number of children	0.14^**^
Likelihood of earthquake within a year	0.10^*^
Sense of preparedness	0.41^***^
Trait anxiety	−0.15^**^
Overall trust in authorities	0.12^*^
Perception of intervention as optimistic	0.19^***^
Perception of intervention as empowering	0.13^*^
Seismic risk	−0.046

Adjusted *p*-value for multiple comparisons is *p* = 0.003

^***^p-value significant at the < 0.001 level (two-tailed) ^**^ < 0.01 ^*^ < 0.05

Given that the preparedness level at the first time point was measured before watching the videos and that the preparedness level at the third time point was measured a few days after the start of the "Iron Swords" war, it was decided to focus the multivariate model on predicting the preparedness index at the second time point (*t* + 14 days). The model was found to be statistically significant (*F* = 9.19, *p* < 0.001). The model explains 30.8% of the variance of the dependent variable. The only predictors of PI were found to be male gender (*β* = 0.19), age (*β* = 0.21), perception of the probability of an earthquake occurring in the next year (*β* = 0.14), and feeling of preparedness at the second measurement point (*β* = 0.28). See full model results in Table 5.

**Table 5:** Results of a multivariate linear regression model for predicting reported preparedness at the second measurement time (*t* + 14 days) (*N* = 297)

Variable	Unstandardized coefficients		Standardized coefficients (*β*)	*t*	*p*-value
	B	Standard Error			
Constant	−1.789	2.493		−.718	.474
Age	.044	.014	.207	3.234	**.001**
Gender	1.340	.460	.189	3.298	**.001**
Number of children	−.030	.122	−.015	−.254	.806
Fear condition	−.050	.449	−.007	−.111	.912
Empowerment condition	.050	.449	.007	.111	.912
Previous experience of an earthquake	−.894	.737	−.070	−.111	.912
Perception of the likelihood of an earthquake in the coming year	.498	.206	.138	2.416	**.016**
Perceiving the video as empowering	−.113	.219	−.031	−.518	.605
Perceiving the video as optimistic	.194	.230	.058	.844	.400
Sense of preparedness at time *t* + 14 days	1.335	.278	.284	4.801	** < .001**
Trait Anxiety	−.020	.019	−.064	−1.056	.292
Overall trust in Authorities	.312	.353	.052	.883	.378

Bold means statistically significant at a *p*-value < 0.05 (two-folds)

Age, number of children, perception of likelihood/empowering/optimistic, sense of preparedness, trait anxiety, and trust—all entered as continuous variables. Gender: female = 0, male = 1; Previous experience of an earthquake: yes = 1, no = 2; Fear condition: yes = 1, no = 0; Empowerment condition: yes = 1, no = 0

## Discussion

### Association between risk communication style and earthquake preparedness in Israel

This study examined the effectiveness of different risk communication methods on household adjustment for earthquakes, focusing on two main approaches (fear-based and empowerment-based). The research findings do not unequivocally support the research hypothesis. While the research findings consistently show, at all measurement points, that participants in the empowerment group reported higher levels of earthquake preparedness, there was no statistical significance to confirm the hypothesis**.** It should be noted that this finding deviates from what has been reported in the literature in recent years. Current studies show that the use of empowering and optimistic risk communication can contribute to and support the creation of behavioral intention and actual behavioral change related to emergency preparedness [48–50].

One possible explanation for the different findings reported in this study is rooted in the manipulation used. Although the development process of the research methodology was based on feedback from an expert panel, the findings show that the intervention in the fear group did not completely isolate the fear element, as many respondents also saw the fear-inducing video as empowering. In this situation, it is possible that the empowerment component in the fear-appeal group may have contributed to closing the gap earthquake preparedness between that group and the empowerment-based group. In other words, the research findings may support the direction of the research hypothesis, but the research groups do not create the necessary distinction to isolate the variables.

The findings of the multivariate analysis, particularly those of the repeated measures ANOVA, shed further light on the research findings. The study findings indicate a borderline significant interaction effect when considering risk communication style and the respondent’s gender. Namely, the results show that women in the empowerment group experienced a greater improvement compared to men. This finding may be explained by the fact that the empowering and optimistic video created for the study features a female protagonist, an active mother who is determined to prepare for an earthquake and protect her family and neighbors. Thus, the video may appeal more to women than to men.

Indeed, previous studies dealing with other scenarios have shown that in emergencies, women (and children) are considered a risk group and that the burden of maintaining the household falls mainly on women [59, 60]. The events of the October 7th attacks further demonstrated the crucial role played by many Israeli women in maintaining and running a household during a crisis when men are mobilized on the fighting front. Therefore, the finding that the video we produced seems to instill a sense of self-efficacy that translates into behavioral intention among women is encouraging and warrants further research. This outcome is particularly important since women report a lower level of preparedness than men. This finding echoes a large body of literature describing gender differences in terms of reported preparedness and perception, which have shown that men, including Israelis, tend to be more confident in their level of earthquake preparedness than women [28, 61–63].

### Correlates of earthquake preparedness in Israel

Correlates of earthquake preparedness behavior has been extensively studied in the last several decades. As early as 2000, Lindell and Perry provided a literature review summarizing the findings of 23 studies [64]. Paton et al. and Becker et al. have expanded these and other findings into actual behavioral models attempting to explain said behavior [22, 65–67]. More recently, Shaffril et al. identified several themes of community preparedness for earthquakes highlighting the importance of community engagement, preparedness training, and strengthening infrastructure to mitigate earthquake threats effectively [68].

The current research findings illustrate additional insights that are worth considering in this regard. First, contrary to what has been reported in the literature for other populations around the world, this study shows no correlation between residing in an area with high seismic risk and threat perception or earthquake preparedness. A possible explanation can be derived from the Victimization model [15, 69], which was originally described in Israel in the context of war. The Victimization model stipulates that a population exposed to a frequent and ongoing threat often resort to denial as an effective coping mechanism ("it won’t happen to me") [15]. Although earthquakes in Israel are not frequent, it is possible that wartime psychology spills over to other hazard types. If that is indeed the case, Israeli risk communicators face a major challenge of overcoming the indifference and habituation effect that delay action by most Israelis until the threat becomes real and imminent [15].

On the other hand, the findings of this study also show that respondents who have experienced a strong earthquake in the past reported higher level of preparedness, which is in line with previous research elsewhere [23, 24, 70]. Similarly, threat perception components, in particular perception of earthquake likelihood, were associated with higher reported preparedness in the current study, in line with previous research [28]. In a 2013 literature review, Lindell concludes that hazard experience, as well as hazard proximity and intrusiveness (frequency of thought and discussion about the hazard), play a significant role in hazard adjustment [71]. More recently, earthquake experience was reported as a significant contributor for households adjustments in Italy [28], South Korea [72], Iran [23], and New-Zealand [24]. It is possible that the seldom nature of earthquakes in Israel allows for classical behavioral models, such as those developed by Paton and Becker et al. [22, 65] to be applicable to the Israeli public in this context.

Moreover, age and number of children are correlated with reported earthquake preparedness. The relationship between age and emergency preparedness is documented in the literature [20, 28]. In general, research in this area suggests that age is related to emergency preparedness for several reasons, including a sense of responsibility to others, especially children [63]. The latter can also explain the association between the number of children and earthquake preparedness. The mixed results observed in this study may suggest that despite spillovers of the Victimization model into Israeli earthquake preparedness behavior, there is still room for intervention due to the infrequent nature of the hazard in the region.

### Recommendation for public health policy

Despite not achieving the statistical significance to support the hypothesis, the study provides insights that support existing literature suggesting that alternative risk messaging approaches to fear tactics are more capable in generating behavioral intent that can lead to sustainable health behavior, in this case, earthquake preparedness. These approaches include the use of optimism and positively framed emotional messages [73], empowerment of individuals and communities to assume a more substantial role in shaping their fate and future [49, 74–76], and re-shaping behavior through socio-normative messaging [73, 77, 78].

It is therefore warranted that policy makers consider that individuals are more inclined to adopt behavioral change in favor of health behavior when the risk messaging is positive, rather than negative. This notion has been widely supported in publications emerging following the COVID-19 pandemic. For example, studies following the COVID-19 pandemic demonstrated the importance of altruism and solidarity in fighting infectious diseases in the USA [79, 80], Germany [44], and Switzerland [45].

Future research is proposed to elaborate and deepen our understanding of the socio-psychological determinants of earthquake preparedness behavior. It is also recommended that future research examine the impact of empowerment on women’s perception of the threat and their willingness to adopt earthquake preparedness behaviors.

### Study limitations

This study has some important limitations. First, like any longitudinal study, this study is also exposed to momentary changes at different measurement points. Between the second and third time points, two significant events occurred that likely affected the results of the study: on September 8, 2023, a strong earthquake occurred in Morocco, and on October 7, 2023, Hamas’ attack in southern Israel unleashed the "Iron Swords" War. Second, the study was conducted online, resulting in a possible selection bias toward respondents with high digital literacy and internet access. The choice for online data collection was made in line with the need for rapid data collection from a nationwide sample. Additionally, the use of online panels has been shown to yield results similar to those collected using other online or offline methodologies [81]. Nonetheless, generalization of the results beyond the adult Hebrew-speaking population in Israel should be done with caution. Third, most of the variables assessed in this study, especially the dependent variable, were based on self-reporting tools. While common practice in the field of research and public opinion polls, this limitation may result in reporting and memory biases**.** Lastly, like many sociological studies, several confounding factors could interfere with the research process and data analysis, not all of which are included in this study.

## Conclusions

The findings showed that the hypothesized superiority of the empowerment condition over the fear condition was not substantiated. However, this might be due to the participants in the fear condition also perceiving that condition as empowering as well. Arguably, the empowerment component in this video contributed to narrowing the gap in preparedness between the group exposed to it and the group exposed to the empowerment messaging, thereby obfuscating the statistical difference between the two groups. Additionally, given that the empowerment video features a female, mother protagonist, it is possible that it resonates more with a female audience, thus highlighting the marginal significance of the gender interaction. Future research should examine the impact of isolated fear and empowerment messages on earthquake preparedness, with a greater emphasis on gender differentiation in this context.

## Data Availability

Data is available from the authors upon reasonable request.
