# Correction: Risk messaging style and its effect on public preparedness for earthquakes: longitudinal intervention-based study

**DOI:** 10.1057/s41271-024-00546-6

**Published:** 2024-12-18

**Authors:** Liel Levy, Moran Bodas

**Affiliations:** Department of Emergency & Disaster Management, School of Public Health, Faculty of Medical and Health Sciences, Tel-Aviv University, Tel-Aviv-Yafo, Israel

**Correction: Journal of Public Health Policy** 10.1057/s41271-024-00534-w

Some funding information was missing from this article and should have read ‘Open access funding provided by Tel Aviv University. This study was supported by the Israeli Ministry of Science, Technology, and Innovation and the National Steering Committee for Earthquake Preparedness (Grant No. 0005462).’

The original article has been corrected.

